# Prospects for chimeric antigen receptor-modified T cell therapy for solid tumors

**DOI:** 10.1186/s12943-018-0759-3

**Published:** 2018-01-12

**Authors:** Erhao Zhang, Jieyi Gu, Hanmei Xu

**Affiliations:** 1 The Engineering Research Center of Peptide Drug Discovery and Development, China Pharmaceutical University, Nanjing, Jiangsu 210009 People’s Republic of China; 2 State Key Laboratory of Natural Medicines, Ministry of Education, China Pharmaceutical University, Nanjing, Jiangsu 210009 People’s Republic of China

**Keywords:** Chimeric antigen receptor T cell, Adoptive cell therapy, Solid tumor, Immunosuppressive microenvironment, Cancer immunotherapy

## Abstract

The potential for adoptive cell immunotherapy as a treatment against cancers has been demonstrated by the remarkable response in some patients with hematological malignancies using autologous T cells endowed with chimeric antigen receptors (CARs) specific for CD19. Clinical efficacy of CAR-T cell therapy for the treatment of solid tumors, however, is rare due to physical and biochemical factors. This review focuses on different aspects of multiple mechanisms of immunosuppression in solid tumors. We characterize the current state of CAR-modified T cell therapy and summarize the various strategies to combat the immunosuppressive microenvironment of solid tumors, with the aim of promoting T cell cytotoxicity and enhancing tumor cell eradication.

## Background

With advances in tumor biology and immunology, cancer immunotherapy has become a new approach for cancer treatment in recent years [1–3]. The adoptive transfer of genetically engineered T cells expressing a chimeric antigen receptor (CAR) specific for tumor antigens, a novel form of cancer immunotherapy, has been remarkably successful in the treatment of some human hematological malignancies, including leukemia and lymphoma [4–8]. The integration of a single chain variable fragment (scFv) and the signaling domain can endow CAR with specificity as well as cytotoxicity in a human leukocyte antigen (HLA)-independent manner [9, 10]. The initial CAR mode comprising the scFv and the CD3ζ signaling domain gives T cells transient activation and cytotoxicity [11]. To improve the cytotoxicity and persistence of CAR-T cells, a costimulatory signaling domain, such as CD28 or CD137 (4/1BB), has been integrated into the intracellular signaling domain in some studies and clinic trials [12, 13].

Despite remarkable success in treating hematological malignancies, particularly in acute lymphoblastic leukemia (ALL) where the T cell therapy achieves high clinical response rates in some trials (e.g., NCT02588456, NCT02535364, and NCT01475058), the adoptive transfer of CAR-T cells has faced a number of challenges for solid tumors [14–18]. Theoretically, modified-T cells have poor homing ability to tumor sites, and a hostile tumor-microenvironment (TME) containing many immunosuppressive cells and other inhibitory factors impairs migrated CAR-T cell cytotoxicity. Although CAR-T cell treatment of solid tumors has not shown promising response, a comprehensive understanding of the multiple barriers seen in the TME is necessary to advance CAR engineering in cancer immunology. In this review, we analyze the factors that limit the application of CAR-T cell therapy in the treatment of solid tumors. We then characterize some new approaches that are being considered to overcome these hurdles, providing guidance for researchers and physicians to effectively fight solid tumors.

## Barriers in the solid tumor microenvironment

### Physical barriers

The extracellular matrix (ECM) in the TME, including proteoglycans and glycopeptidases, has multiple effects on the biological behaviors of tumors and the remodeling of the immune system. Some studies have shown that proteins in ECM that are nonstructural matrix proteins, such as heparan sulfate proteoglycans (HSPGs), have a major role in the maintenance of tumor cell proliferation and migration. [19, 20]. T cells attacking stroma-rich solid tumors have little ability to penetrate and aggregate in tumor sites, resulting in lower antitumor activity [21]. Therefore, improving the capacity of modified-T cells to specifically degrade the ECM in stroma-rich solid tumors, yet without compromising their cytotoxicity, would enhance their antitumor activity.

### Immunosuppressive cells and secreted cytokines

In the TME, immune suppression is mainly mediated by immunosuppressive cells, including regulatory T cells (Tregs), myeloid-derived suppressor cells (MDSCs), and M2 macrophages. It has become clear that these cells and released cytokines, such as transforming growth factor-β (TGF-β) and interleukin (IL) 10, inside solid tumors seriously dampen the efficacy of infused CAR-T cells.

#### Tregs

Tregs play an important role in the regulation of immune responses, including CD4^+^CD25^+^ Tregs and type 1 Tregs. TGF-β is essential for CD4^+^CD25^+^ Treg function while Foxp3, a regulator of its transcription factor, is highly expressed on Tregs [22]. Type 1 Tregs exert their suppressive activity through the secretion of the cytokine IL-10. In general, Tregs are enriched at the site of inflammation and tumors where they modulate the immune reaction via various mechanisms [23, 24]. Activated Tregs can directly eliminate excessive T cells by rapidly taking up IL-2, resulting in a lack of sufficient effector cells against malignant cells [25]. In addition, these inhibitory cells can produce many immunomodulatory cytokines for the suppression of T cell activity, such as TGF-β and IL-10 [26].

#### MDSCs

MDSCs, a major component of the immunosuppressive cells, negatively regulate immune responses against tumour progression and metastasis, impairing antitumor immunity [27, 28]. MDSCs mediate their suppression of T cell activity through a combination of major factors, such as inducible nitric oxide synthase (iNOS), arginase catalyze I (ARG1), cyclooxygenase-2 (COX-2), prostaglandin E2 (PGE2), TGF-β, IL-10, and Tregs. Additionally, the presence of MDSCs is associated with the growth of cancer cells. Therefore, inhibiting MDSCs function has been shown to improve antitumor immune responses in the TME [29, 30].

#### Tumor associated macrophages

M2 macrophages, termed tumor associated macrophages (TAMs), play a pivotal role during the proliferation, invasion, and metastases of tumor cells via expressing soluble proteins and cytokines, such as IL-10, matrix metalloproteinases (MMPs), urokinase-type plasminogen activator (uPA), basic fibroblast growth factor (bFGF), vascular endothelial growth factor (VEGF), platelet-derived growth factor (PDGF), granulocyte-macrophage colony stimulating factor (GM-CSF), and migration stimulating factor (MSF) [31, 32]. Expressed IL-10 efficiently suppresses the activation of cytotoxic T lymphocytes (CTLs) and natural killer (NK) cells, resulting in the rapid proliferation of tumor cells. Excessive M2 macrophages release MMP2 and MMP9 that can specifically degrade the extracellular matrix, thereby promoting the migration of tumor cells and tumor stromal cells [33]. In addition, M2 macrophages can consume L-arginine to produce ornithine and polyamine that promote tumor cell proliferation [34].

### Immunosuppressive inhibitor receptor

#### Cytotoxic T lymphocyte-associated antigen 4

In general, T cell activity is regulated through both the activation signal pathway and the suppression signal pathway, resulting in a balanced activity of T cells in a healthy body. The activation of T cells by antigen stimulation signaling and CD28 costimulation is followed by proliferation and functional differentiation. When the activity is excessive, an inhibitory program that is mediated by cytotoxic T lymphocyte-associated antigen 4 (CTLA4) binding with B7 molecules will eventually terminate T cell function. CTLA4, a homolog of CD28, exerts an inhibitory signal to T cells, and then makes them with an inactive state [35]. Theoretically, CTLA4 recruits a phosphatase to the CD3ζ domain of T cell receptors (TCRs), thereby attenuating T cell function by the dephosphorylation of immunoreceptor tyrosine-based activation motifs (ITAM) [36]. In addition, some studies have demonstrated that TCR activation of T cells initiates the phosphorylation of the CTLA4 intracellular domain, termed the immunoreceptor tyrosine-based inhibitory motif (ITIM), and prolongs the CTLA4 expression on the activated T cell surface [37, 38]. Based on the principle of CTLA4 function, approaches that reduce CTLA4 expression or inhibit its activity have been proposed for cancer immunotherapy.

#### Programmed death 1

Another important immunologic target for cancer immunotherapy after CTLA4 is programmed death 1 (PD1), a cell surface receptor that belongs to the immunoglobulin superfamily expressed only on activated T lymphocytes [39]. Structurally, the mature PD1 protein comprises an extracellular recognition domain targeting its ligands (PDL1 or PDL2) presented on tumor cells and a transmembrane region followed by an intracellular tail [40]. Functionally, PD1 plays an important role in suppressing T cell activity due to the cytoplasmic tail having two phosphorylation sites, an ITIM and an immunoreceptor tyrosine-based switch motif (ITSM), that both negatively regulate T-cell receptor signals [41]. Based on CTLA4 and PD1 functions in different suppression pathways, a combination therapy of PD1 antibody and CTLA4 antibody, therefore, has emerged as an effective approach to combat cancers via checkpoint inhibition [42].

#### Other immune checkpoints

Although CTLA4 and PD1 pathways negatively regulate the immune response of T lymphocytes against cancers, they only represent the tip of the iceberg of potential targets that can decrease antitumor responses in the immune system. With the development of T cells for cancer immunotherapy, researches have revealed other immune checkpoints that significantly suppress the immune response, such as lymphocyte-activation gene 3 (LAG3), T-cell immunoglobulin mucin 3 (TIM3), and V-domain Ig suppressor of T cell activation (VISTA) [43–45]. The protein LAG3, an exhaustion marker, is wildly expressed on activated T cells and other immune cells, and it is an important inhibitory receptor that binds major histocompatibility complex class II (MHC II) with higher affinity than CD4, preventing antitumor immune responses [46]. TIM3, a receptor protein expressed on T cells, initiates the TIM3/Galectin-9 signaling pathway, which can inhibit lymphocyte responses and promote apoptosis [47]. VISTA, a potent negative regulator receptor expressed on hematopoietic cells and leukocytes, reduces the activity of T cell and the production of cytotoxic cytokines [48].

### Intratumoral inhibitory factors

For solid tumors, a hostile microenvironment is characterized by poor oxygen (O_2_) and nutrient supply [49–51]. In general, cancer cells exhibit unregulated growth even with nutrient scarcity and O_2_ deprivation due to the constitutive activation of growth-promoting pathways dependent on unsaturated fatty acid catabolism [52]. Additionally, a lower pH of the tumor extracellular environment is frequently observed as a result of high hydrogen ions levels derived from lactic acid and carbonic acid [53]. Glycolysis produces a large amount of lactic acid under anaerobic conditions, and carbonic acid is synthesized from CO_2_ and H_2_O in the presence of a carbonic anhydrase (CA) expressed on tumor cells, which maintain an acidic microenvironment.

### Lack of chemokine receptors

The primary obstacle when using CAR-modified T cells is infiltration into the solid tumor bed [54]. Some solid tumors secrete chemokines, such as CXCL12 and CXCL5, which are not conducive to T cells penetration into the intratumoral site [55]. In contrast, T cells lack some chemokine receptors that adequately match the chemokine signature secreted from solid tumors [56]. Some reports show that, although chemokines such as C-C chemokine ligand 2 (CCL2) which belongs to the C-C chemokine family, are highly secreted by solid tumors, the corresponding chemokine receptors (CCR), such as CCR2b and CCR4, that can specifically bind CCL2 are minimally expressed on T cells, resulting in a lack of homing to the tumor site [57].

### Tumor antigen heterogeneity

To the best of our knowledge, CAR-T cells identify and target surface antigens presented on cancer cells through scFv structures that compose the variable domains of the light and heavy chains derived from monoclonal antibodies. The membrane protein CD19 is homogenous and widely expressed on almost all B-cell hematologic malignancies, resulting in encouraging responses from CD19-specific CAR-T cell therapy [58–61]. However, for solid tumors, the application of CAR-T cell therapy is severely limited due to a shortage of tumor-specific antigens and heterogeneity [62]. However, solid tumors are vulnerable to relapse after treatment with any therapy approach, resulting in cancer being known as a wound that does not heal.

### Other immunosuppressive factors in human immune system

#### CD47

In the human immune system, macrophages exert phagocytosis based on a balance between phagocytic (“eat me”) and antiphagocytic (“do not eat me”) signals on target cells. The antiphagocytic signal depends on the interaction between CD47 expressed on target cells and signal regulatory protein α (SIRPα) presented on macrophages, leading to the phosphorylation of ITIMs on SIRPα. CD47, a transmembrane protein, has been found to be overexpressed in many tumor cells [63–66]. In general, the CD47-SIRPα-mediated antiphagocytic signal allows tumor cells to evade immune surveillance [67–69]. Therefore, to enhance the ability of the immune system against various cancers, CD47 can be used as a novel target for cancer immunotherapy.

#### CD73 and adenosine

To date, eliminating solid tumors by CAR-T cell therapy has been largely unsuccessful due to adenosine production. Adenosine is generated from extracellular adenosine monophosphate (AMP) by the ectoenzyme CD73 (ecto-5′-nucleotidase) expressed on tumor cells or immunosuppressive cells [70]. The produced adenosine significantly inhibits modified-T cell cytotoxicity through the activation of adenosine 2A receptors (A_2A_R), resulting in T cells that do not effectively function against solid tumors [71, 72].

#### Indoleamine 2,3-dioxygenase

Indoleamine 2,3-dioxygenase (IDO) is an immunosuppressive target that helps create a tolerogenic milieu in tumor sites, mainly by suppression of T cell cytotoxicity and enhancement of Treg-mediated immunosuppression [73, 74]. In addition, IDO can suppress antitumor activity of T cells through degradation of tryptophan, a key amino acid required for T cell activity [75, 76]. A series of tryptophan metabolites in the kynurenine pathway suppresses T cell activity and induces T cell apoptosis, also affecting NK cell function [77].

**Fig. 1:**
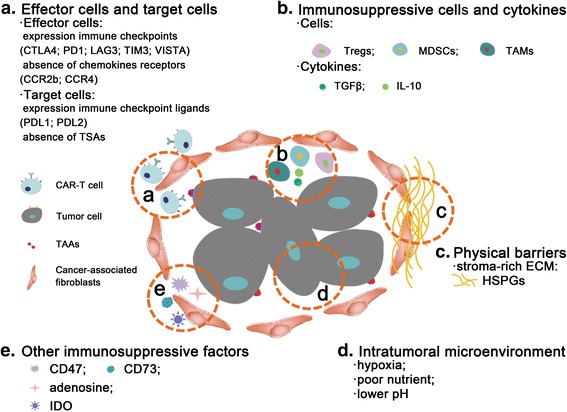
Immunosuppressive microenvironment in solid tumors. **a** Effector cells and target cells. Activated T cells often express some immune checkpoints that rapidly neutralize the antitumor activities, such as CTLA4, PD1, LAG3, TIM3, and VISTA, while the target cells express some immune checkpoint ligands, such as PDL1 or PDL2. In general, T cells lack some receptors, such as CCR2b or CCR4. In addition, CAR-T cell therapy is limited in solid tumors due to the paucity of tumor-specific antigens. **b** Immunosuppressive cells and cytokines. In the solid TEM, some immunosuppressive cells (e.g., Tregs, MDSCs, and TAMs) and released immunosuppressive cytokines (e.g., TGFβ and IL-10) significantly inhibit CAR-T cell cytotoxic function. **c** Physical barriers. Stroma-rich solid tumors have an abundance of ECM (e.g., HSPGs) that effectively inhibits the penetration and aggregation of T cells. **d** Intratumoral microenvironment. The intratumoral microenvironment is associated with hypoxia, nutrient starvation, and acidosis derived from elevated lactate generation. **e** Other immunosuppressive factors. CD47 allows tumor cells to evade immune surveillance mediated by CD47-SIRPα-mediated antiphagocytic signaling. Adenosine generated from extracellular AMP by the ectoenzyme CD73 results in unsuccessful application of CAR-T cell therapy. IDO contributes to tumor-induced tolerance through the degradation of tryptophan

In summary, CAR-modified T cells are faced with numerous barriers when acting agains solid tumors with blunt immune-surveillance, achieving a poor response in some clinical trials (Fig. 1). With further research into tumor biology and immunology, some strategies have been developed to overcome these obstacles, allowing for clinical application of CAR-T cell therapy. Next, we describe these approaches to improve the TME and enhance T cell functions according to their mechanisms and their applications.

## Approaches to improve the CAR-T cell efficacy

### Selecting tumor-specific antigens

**Fig. 2:**
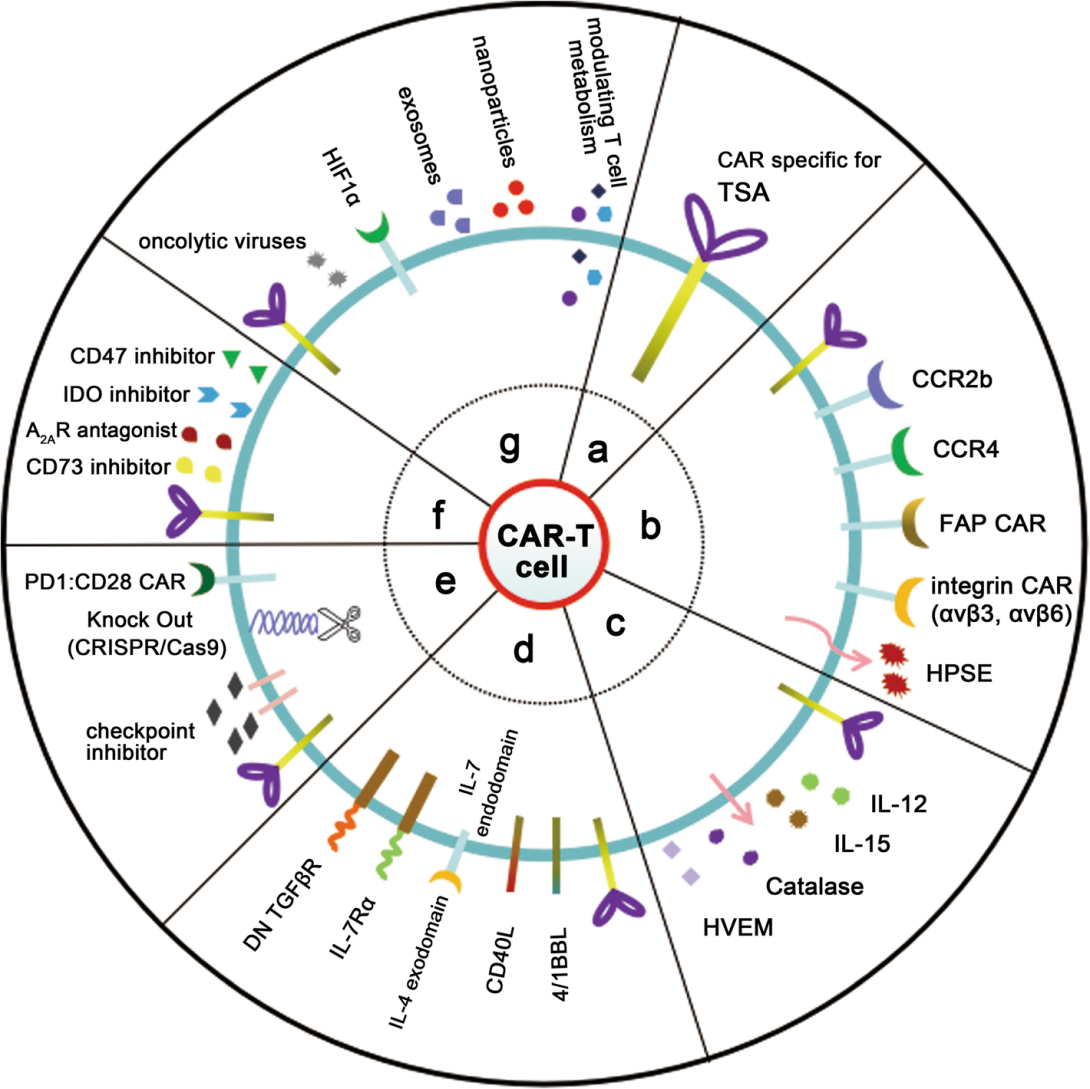
Novel strategies to enhance the efficacy of CAR-T cell therapy for solid tumors. **a** CAR-T cells targeting tumor specific antigens. **b** Infiltration and homing of CAR-T cells. CAR-modified T cells express a chemokine receptor (e.g., CCR2b or CCR4), increasing their ability to move to the tumor site. FAP-specific CAR-T cells can migrate to the tumor bed, similar to integrin αvβ3 (or αvβ6)-specific CAR-T cells. Heparanase expression enhances CAR-T cell infiltration and antitumor activity. **c** CAR-T cells secreting cytokines or enzymes. To resist a hostile environment, T cells have been engineered to express cytokines, such as IL-12 or IL-15. CAR-T cells are endowed with the catalase to overcome abundant ROS. CAR-T cells carrying soluble HVEM also enhance therapeutic activity against lymphomas. **d** CAR-T cells expressing receptors. CAR-T cells expressing costimulatory receptors (e.g., 4/1BBL or CD40L) significantly enhance T cell activity. CAR-T cells expressing a hybrid receptor comprising an IL-4 exodomain and IL-7 endodomain convert the immunosuppressive response to immune action by targeting IL-4. CAR-T cells expressing IL-7Rα specific for IL-7 have improved response in solid tumors. T cells with a dominant-negative TGFβ (DN TGF-βR) response to TGFβ. **e** Immune checkpoint therapy. CAR-T cell therapy can be combined with the blockage of immune checkpoints using monoclonal antibodies or the CRISPR/Cas9 system. Additionally, PD1:CD28 CAR comprising the PD1 exodomain and CD28 endodomain enhance T cell activity. **f** Other blockage therapies. Blockage of soluble tumor suppressive mediators in the solid tumor milieu (e.g., CD73, A_2A_R, IDO, or CD47) enhance CAR-T cell function. **g** Combination therapies. CAR-T cells can be combined with other antitumor strategies, such as oncolytic viruses, HIF-CAR, exosomes, nanoparticles, and modulating T cell metabolism

The antigenic specificity of the CAR structure directly determines the precise activity and safety of modified-T cells. However, for solid tumors, the heterogeneity of tumor antigens results in invalid immune surveillance, and thereby a refractory and relapsed tumor. Some researchers have attempted to find markers of cancer stem cells, which are considered to be tumor-specific antigens in the early stage of tumor differentiation [78, 79]. The identification of a tumor-specific antigen provides a powerful tool to develop targeted therapies for solid tumors (Fig. 2a).

### Infiltration and homing of CAR-T cells

#### Chemokine receptors

To enhance T cell homing to tumor cells, some chemotactic manipulations endow T cells with a strong ability to target solid tumors. A combination of chemokines secreted from tumor cells and corresponding chemokine receptors presented on genetically modified-T cells may facilitate a greater proportion of T cells to arrive at the solid tumor site (Fig. 2b). For malignant pleural mesotheliomas, T cells genetically modified to simultaneously express CCR2b and a CAR specific for mesothelin increase their ability to migrate to tumor sites and eliminate target cells. In addition, other groups have described a similar CAR methodology with augmented trafficking and antitumor efficacy, such as using CD30-specific CAR-T cells expressing CCR4 in Hodgkin’s lymphoma and GD2-specific CAR-T cells expressing CCR2b for neuroblastoma [80, 81]. In these studies, CAR T cells modified with a functional chemokine receptor have adequate tumor localization and significant antitumor efficacy in vivo.

#### Fibroblast activation protein

Previous reports have demonstrated that cancer-associated fibroblasts (CAFs) facilitate angiogenesis, playing a prominent role in the progression and metastasis of solid tumors [82, 83]. Therefore, CAFs are a key determinant in the malignant progression of cancer and represent an important target for cancer therapies. Schuberth and colleagues developed T cells with a CAR specific for a fibroblast activation protein (FAP), which is abundantly expressed on CAFs, and obtained an encouraging response in vivo, leading to CAF-CAR-T cells being used in an ongoing clinical trial (NCT01722149) (Fig. 2b) [84]. In summary, targeting CAFs as a therapeutic strategy is an intriguing approach to effectively eradicate cancer.

#### Integrin

For solid tumors, modified-T cells lack the ability to infiltrate into the tumor parenchyma, resulting in limited their effectiveness in vivo. In general, integrin αvβ3 is highly expressed on the surfaces of endothelial cells of tumor neovasculature in solid tumors. Fu and colleagues, therefore, put forward a promising strategy in which T cells are genetically engrafted with an echistatin-containing CAR (T-eCAR) specific for αvβ3 integrin (Fig. 2b) [85]. The injection of T-eCAR cells effects tumor neovasculature, leading to significant tumor shrinkage, yet has no significant off-target effect on blood vessels in normal tissues. In addition, Whilding and colleagues developed a novel CAR modality specific for αvβ6 integrin expressed on ovarian, breast, and pancreatic tumors [86].

#### Heparanase

Solid tumor-mediated immunosuppression may play an important role in impeding CAR-T cell activity attributed to the HSPGs-rich ECM, inhibiting the ability of modified-T cells to accumulate in tumor sites. Therefore, a strategy to degrade HSPGs around stroma-rich solid tumors is an effective way to enhance the antitumor activity of CAR-redirected T cells, such as by enzymatic degradation of heparanase (HPSE) (Fig. 2b) [87]. Caruana and colleagues reported that HPSE expression enhances CAR-redirected T cell infiltration and antitumor activity. In a neuroblastoma model, their results demonstrated that inducing the expression of HPSE in LET-T cells endowed with a GD2-specific CAR enhanced their capacity to degrade the ECM [88].

### Co-expressing immune factors or enzymes

To further improve the ability of T cells to resist the evil TME, T cells have been engineered to constitutively express some cytokines, such as IL-12 or IL-15, and secrete some enzymes, such as catalase (Fig. 2c).

#### IL-12

The immunomodulatory cytokine IL-12 promotes the rapid reversal of immune suppression and plays an important role in the activities of T lymphocytes and other immune cells, emerging as a potent inducer of antitumor immunity [89]. For instance, Pegram and colleagues reported that CD19-targeted CAR-T cells secreting IL-12 eliminate systemic tumors and achieve optimal antitumor response [90]. In addition, CAR redirected T cells delivering IL-12, termed TRUCKs, not only enhance the activation and cytotoxicity of modified-T cells, but also result in a Th1 polarization state that attracts endogenous T cells and other innate immune cells to eradicate cancer cells [91, 92].

#### IL-15

Some studies have shown that the cytokine IL-15 is a potent stimulant of memory CD8^+^ T cells and NK cells by binding the IL-2 receptor β-common γ chain (IL2Rβγc) complex [93]. Hsu and colleagues demonstrated that IL-15 plays an important role in the trafficking of CD8^+^ T cells in vivo [94]. Therefore, IL-15-expressing T-cells could be used as an effective weapon to eliminate target cells within the immunosuppressive microenvironment.

#### Catalase

In solid tumors, infused-T cells often encounter abundant reactive oxygen species (ROS), a hallmark of the tumor milieu, which can impair the antitumor activity of modified-T cells [95]. Ligtenberg and colleagues developed a novel T cell modality (termed CAR-CAT T cell) that is endowed with a CAR specific for catalase, which catalyzes hydrogen peroxide to water and oxygen [96]. These engineered T cells could reduce the oxidative state of infiltrated tumors and preserve their antitumor activity by metabolizing hydrogen peroxide and reducing oxidative stress-mediated suppression.

#### HVEM

Herpesvirus entry mediator (HVEM), known as tumor necrosis factor receptor superfamily member 14 (TNFRSF14), is among the most frequently mutated genes in germinal center lymphomas (GCL) [97, 98]. Loss of HVEM usually drives the development of GCL in vivo and induces a tumor-supportive microenvironment, resulting from the disruption of the interactions between HVEM and BTLA (B and T lymphocyte attenuator) receptors [99, 100]. Boice and colleagues engineered CD19-targeted CAR-T cells carrying soluble HVEM (solHVEM) to locally and continuously enhance therapeutic activity against lymphomas by release of solHVEM [101].

### Modifying some related receptors

In general, T cells expressing a CAR structure have a promising immunotherapeutic effect on B cell malignancies [102, 103]. In recent years, the design of novel CARs modified with costimulatory receptors or cytokine receptors has resulted in important effects on T cell activity, including proliferation and cytotoxicity.

#### 4/1BBL

Some recent reports have revealed that second-generation CARs constitutively expressing ligands for CD28 or 4/1BB (CD80 or 4/1BBL, respectively) enhance T cell activity in vivo [104]. Stephan and colleagues suggested that the 4/1BB-4/1BBL signaling pathway may more effectively enhance CAR modified-T cell activation and cytotoxicity, potentially in a bidirectional manner with direct signaling within the T cells themselves and indirect triggering of other immune cells expressing the 4/1BB domain in the TME (Fig. 2d) [105].

#### CD40L

Cancer immunotherapy requires effective destruction of the immunosuppressive TME. Marigo and colleagues demonstrated that the CD40/CD40L axis is necessary for tumor rejection mediated by CD8^+^ T cells in the presence of tumor necrosis factor (TNF) derived from dendritic cells (DCs) [106]. Therefore, we envisioned that tumor elimination will be achieved by a combination of CAR-modified T cells with CD40/C40L pathway modifications (Fig. 2d).

#### IL-7Rα

Cytokine IL-7 is a common γ chain cytokine that is essential for the expansion of naive and memory T lymphocytes [107]. To maintain T cells with high efficacy, CTLs can be genetically modified to express IL-7Rα that can specifically bind to IL-7 (Fig. 2d). Vera and colleagues reported that the expression of IL-7Rα consistently promotes CTL activity, including activation and proliferation, in response to IL-7, and provides antitumor activity without impairing the antigen specificity or dependency of modified-CTLs [108].

#### DN TGF-βR

There are many immunosuppressive cytokines secreted by tumor cells in the TEM, including TGF-β, which markedly inhibits tumor-specific cellular immunity. Based on this mechanism, reducing the expression of TGF-β receptor (TGF-βR) presented on T cells is an effective strategy to surmount TGF-β-induced immune suppression [109]. Bollard and colleague demonstrated that EBV-specific T-cells endowed with a dominant-negative TGF-βR (DN TGF-βR) can resist the inhibitory effects of TGF-β, resulting in T cells with longer survival (Fig. 2d) [110].

### Blockage of the immune checkpoints

Previous reports have revealed that some tumors, especially solid cancers, employ a variety of countermeasures to impair the cytotoxicity of T cells, including the expression of immune checkpoints expressed on T cells, such as PD1, CTLA4, LAG3, TIM3, or VISTA. These inhibitory receptors expressed on activated T cells have a high affinity for their ligands present on target cells, therefore the inhibitory receptor/ligand pathway is a powerful brake on T cell efficacy. Some technologies have been developed to overcome this obstacle, including monoclonal antibodies (mAb) and gene editing (Fig. 2e).

For mAb immunotherapy, clinical trials associated with the blockade of immune checkpoints have demonstrated that mAbs can reverse T cell exhaustion and restore antitumor immunity, significantly promoting tumor cell eradication, such as anti-PD1 mAb (NCT02838823, NCT02857166, NCT02836795), anti-CTLA4 mAb (NCT00610857, NCT02156804, NCT03203876), anti-LAG3 mAb (NCT02658981, NCT01968109, NCT02061761), anti-TIM3 mAb (NCT02817633), and anti-VISTA mAb (NCT02671955).

In addition, a non-viral approach to reprogram primary human T cells by disruption of immune checkpoints has been developed with the CRISPR/Cas9 technology. Su and colleagues demonstrated that gene knockout of PD1 by electroporation of plasmids encoding sgRNA and Cas9 results in significant reduction of PD1 expression, up-regulation of cytokine production, and enhanced cytotoxicity [111]. Shi and colleagues demonstrated that CTLs with knocked-out CTLA4 by CRISPR/Cas9 system significantly repress tumor growth and improve tumor eradication [112]. Therefore, a two-in-one approach including CAR-T cells and checkpoint blockade (PD1 or CTLA4) is potentially useful to improve the efficacy of T cells.

Moreover, to reverse the PDL1-mediated T cell exhaustion, Prosser and colleagues provided a novel engineering strategy in which the PD1 was converted to a T cell costimulatory receptor by exchanging its transmembrane and cytoplasmic domains with that of CD28 (Fig. 2e) [113]. In their research, tumor PDL1 significantly co-stimulated primary human T cells expressing a PD1:CD28 chimeric receptor, resulting in augmentation of cytokine secretion and increased cytotoxicity.

### Other blockage therapies

#### CD73 inhibition and A_2A_R antagonist

Based on adenosine-derived immune suppression around solid tumors, there are two broad strategies to enhance CAR-T cell efficacy (Fig. 2f). One approach is to block the interaction between adenosine and A_2A_R by A_2A_R antagonists, profoundly increasing CAR-T cell cytotoxicity. For instance, Beavis and colleagues showed that human epidermal growth factor receptor 2 (HER2)-specific CAR-T cells had high efficacy against melanoma tumor cells expressing HER2 in the presence of an A_2A_R antagonist [114]. As a result of this preclinical work, a clinical trial (NCT02655822) is ongoing using A_2A_R antagonists against various solid tumors, such as non-small cell lung cancer, malignant melanoma, renal cell cancer, triple negative breast cancer, colorectal cancer, bladder cancer, and metastatic castration-resistant prostate cancer. Another approach is to generate an mAb specific for CD73 within the tumor milieu, resulting in the reduction of CD73-derived adenosine [115, 116]. In a current phase I clinical study (NCT02503774), the use of anti-CD73 mAb alone or in combination with anti-PDL1 mAb in patients with advanced solid tumors is showing promising response [117].

#### IDO inhibition

IDO can inhibit T cell efficacy by reducing the concentration of tryptophan and activating Tregs through kynurenine production, which contributes to immunosuppressive microenvironment [118, 119]. In a recent study, the inhibition of the IDO pathway showed significant clinical activity in patients with various cancers (Fig. 2f) [120]. Therefore, there is great interest in combining IDO inhibitors with CAR-T cell therapy for the treatment of solid tumors in future studies.

#### CD47 inhibition

For many malignancies, abundant CD47 expression is associated with poor patients survival. Some studies have reported that macrophage phagocytosis of tumor cells mediated by anti-CD47 antibodies is a major effector of the immune system. Additionally, Liu and colleagues demonstrated that anti-CD47-mediated tumor rejection required the activation of T cells (Fig. 2f) [121]. Hence, CAR-T cell therapy combined with CD47 inhibition could improve tumor elimination by enhancing the immune system.

### Combination strategies for CAR-T therapy

T cells engineered to express CAR in adoptive transfer therapies have thus far shown significant promise in treating some hematological cancers. The clinical efficacy of CAR-T cell therapy in treating solid tumors, however, is negatively affected by a hostile TME. With advancements in tumor research, combination therapies, including T cells or modified-T cells with other antitumor strategy, should have high efficacy for solid tumor eradication (Fig. 2g).

#### Oncolytic viruses

Oncolytic viruses (OVs) have the capacity to selectively infect and promote tumor cell lysis and impact tumor growth via reducing tumor vasculature, while avoiding healthy tissue damage. In addition, these viruses have been shown to induce robust immune responses, thus potentiating antitumor responses to decrease the tumor burden [122, 123]. VanSeggelen and colleagues demonstrated that loading CAR-engineered T cells with low doses of virus does not impact CAR expression and activity [124]. Combining CAR-T cells with OVs represents a novel strategy to increase the efficacy of cancer treatments. In addition, Nishio and Dotti proposed to improve the migration and survival of CAR-modified T cells in solid tumors by combining them with armed OVs expressing the chemokine RANTES and the cytokine IL-15 [125]. In their experiments, the modified OVs significantly enhanced the trafficking and infiltration of CAR-T cells, thereby promoting tumor eradication.

#### HIF-CAR

CAR-T cell therapies raise concerns associated with “on-target, off-tumor” effects, particularly in healthy tissues expressing the targeted antigen. To effectively discriminate between healthy tissue and cancer cells, CAR-modified T cells have been endowed with other receptors specific for TME signals. As hypoxia is a hallmark of solid TME, an interesting strategy was exploited for CAR-T cell therapy by targeting hypoxia [126, 127]. For instance, Juillerat and colleagues constructed a novel CAR modality, termed HIF-CAR, that fuses an oxygen sensitive sub-domain of hypoxia-inducible factor 1-alpha (HIF1α), resulting in a CAR sensitive to a hypoxic environment [128]. HIF-CAR-T cells have specific cytotoxicity for solid tumors in the presence of both a tumor antigen and a TME signal (hypoxia), providing a basic approach for using multi-chain modified-CAR as a platform to create the next generation of CAR-T cells.

#### Exosomes

Exosomes play an important role in intercellular communication and mediate a cell-cell communication with a novel mechanism [129]. In addition, exosomes act as transport carriers for delivering functional proteins secreted from donor cells into the extracellular milieu, thereby improving the interaction between effector cells and target cells. Based on the properties of exosomes, CAR-T cell-derived exosomes have been used to attack tumors in place of immune cells, allowing for controlled cytotoxicity and avoiding some adverse effects of CAR-T cell-mediated immunotherapy. Additionally, exosomes derived from CAR modified-T cells pave the way for cancer immunotherapy against glioblastoma due to their nanoscaled size. Tang and colleagues created a platform, including CAR-T cells and CAR-T cell-derived exosomes, for the treatment of some tumors [130]. Kamerkar and colleagues applied exosomes to effectively facilitate therapeutic targeting of oncogenic KRAS in pancreatic cancer and significantly increase overall survival [131].

#### Nanoparticles

With the development of nanotechnology, nanomedicine-based immunotherapy has been used to elicit specific antitumor responses, providing a new avenue to eliminate solid tumors. Nanoparticle (NP) can be used as a delivery vehicle for releasing antitumor drugs or genes coding CAR structures, and also as immune adjuvants to stimulate the host’s immune system [132–135]. For instance, Yuan and colleagues constructed a multivalent bi-specific nanobioconjugate engager (mBiNE) derived from colloidal-NPs to promote immune-mediated tumor eradication [136]. mBiNE can excit a durable antitumor response for HER2-expressing tumors through a pro-phagocytosis signaling mediated by calreticulin. Thus, cancer immunotherapy based on mBiNE provides a new strategy to stimulate both innate and adaptive immunity. Smith and colleagues programmed T cells with tumor-recognizing capabilities in which DNA-carrying NPs facilitated T cells to express CD19-targeting CAR, thereby providing long-term tumor remission [137]. They functionalized the targeted NPs to specifically target the T cells by coupling with anti-CD3e f(ab’)2 fragments, and loaded the NPs with a vector encoding CD19-specific CAR. In summary, they established, for the first time, a practical and simplified way to modify T cells with the ability to express CD19-specific CARs by polymeric NPs, providing a new form of active immunotherapy suitable for clinical trials.

#### Modulating T cell metabolism

The metabolic activity of T cells is intimately linked to their efficacy against cancer. Therefore, regulating the state of T cells in the presence of exogenous active agonists would be an effective strategy to improve tumor eradication. For instance, Geiger and colleagues discovered that elevating the intracellular concentration of L-arginine modulates T cell metabolism, including a shift from glycolysis to oxidative phosphorylation, and promotes the generation of central memory-like cells, thereby enhancing the antitumor activity [138]. Yang and colleagues engineered a new mechanism of T cell cytotoxicity of by which CD8^+^ T cells can be potentially improved by modulating cholesterol metabolism [139]. Scheffel and colleagues reduced T cell death with the addition of the antioxidant N-acetyl cysteine (NAC), offering a potential method to improve the quality and therapeutic efficacy of adoptive T cells [140].

Taken together, the above-mentioned studies offer preclinical proof-of-concept for modulating the metabolism of therapeutic T cells for tumor eradication.

## Conclusions

The great success of CAR T cell therapy in some clinical trials against hematological malignancies has provided a framework to drive this modality further to treat solid tumors with immunosuppressive factors. In recent years, the characteristics of the immunosuppressive environment have been described in detail, producing new approaches to overcoming obstacles and enhancing tumor eradication by CAR-T cell therapy. In this review, we have given a brief overview of the challenges against immunologic surveillance in the solid TME, and discussed some promising approaches to enhance CAR-mediated T cell efficacy by optimizing the CAR structure and improving tumor-induced immunosuppression. For the application of CAR-T cell therapy against solid tumors, additional strategies must be explored to modify T cells to achieve sustained cytotoxicity, and to remodel TME to further enhance tumor destruction. Finally, we envision that combination immunotherapies, including enhanced T cell activity (such as infiltration and specific targeting), and improvement of the solid intratumoral environment (such as clearing physical and biochemical barriers) will become effective means to treat solid tumors.

## Abbreviations

A_2A_R: Adenosine 2A receptor
ALL: Acute lymphoblastic leukemia
AMP: Adenosine monophosphate
ARG1: Arginase catalyze I
bFGF: Basic fibroblast growth factor
BTLA: B and T lymphocyte attenuator
CA: Carbonic anhydrase
CAFs: Cancer-associated fibroblasts
CAR: Chimeric antigen receptor
CAR-CAT: CAR co-expression catalase
CCL2: C-C chemokine ligand 2
CCR: Corresponding chemokine receptors
COX-2: Cyclooxygenase-2
CTLA 4: Cytotoxic T lymphocyte-associated antigen 4
CTLs: Cytotoxic T lymphocytes
DCs: Dendritic cells
DN TGF-βR: Dominant negative TGF-βR
ECM: Extracellular matrix
FAP: Fibroblast activation protein
GCL: Germinal center lymphomas
GM-CSF: Granulocyte-macrophage colony stimulating factor
HER2: Human epidermal growth factor receptor 2
HIF1α: Hypoxia-inducible factor 1-alpha
HLA: Human leukocyte antigen
HPSE: Heparanase
HSPGs: Heparan sulfate proteoglycans
HVEM: Herpesvirus entry mediator
IDO: Indoleamine 2,3-dioxygenase
IL: Interleukin
IL2Rβγc: IL-2 receptor β-common γ chain
iNOS: Inducible nitric oxide synthase
ITAM: Immunoreceptor tyrosine-based activation motifs
ITIM: Immunoreceptor tyrosine-based inhibition motif
ITSM: Immunoreceptor tyrosine-based switch motif
LAG3: Lymphocyte-activation gene 3
mAb: Monoclonal antibody
mBiNE: Bi-specific nanobioconjugate engager
MDSCs: Myeloid-derived suppressor cells
MHC II: Major histocompatibility complex class II
MMPs: Matrix metalloproteinases
MSF: Migrate stimulate factor
NAC: N-acetyl cysteine
NK: Natural killer
NP: Nanoparticle
OVs: Oncolytic viruses
PD1: Programmed death 1
PDGF: Platelet-derived growth factor
PDL1: PD ligand 1
PDL2: PD ligand 2
PGE2: Prostaglandin E2
ROS: Reactive oxygen species
scFv: Single chain variable fragment
SIRPα: Signal regulatory protein α
solHVEM: Soluble HVEM
TAMs: Tumor associated macrophages
TCR: T cell receptor
T-eCAR: Echistatin-containing CAR T
TGF-β: Transforming growth factor-β
TGF-βR: TGF-β receptor
TIM3: T-cellimmunoglobulin mucin 3
TME: Tumor microenvironment
TNF: Tumor necrosis factor
TNFRSF14: Tumor necrosis factor receptor superfamily member 14
Tregs: Regulatory T cells
uPA: Urokinase-type plasminogen activator
VEGF: Vascular endothelial growth factor
VISTA: V-domain Ig suppressor of T cell activation
